# The Multidimension of Malnutrition among School Children in a Rural Area, South Africa: A Mixed Methods Approach

**DOI:** 10.3390/nu14235015

**Published:** 2022-11-25

**Authors:** Perpetua Modjadji, Sphiwe Madiba

**Affiliations:** 1 Department of Public Health, School of Health Care Sciences, Sefako Makgatho Health Sciences University, Ga-Rankuwa MEDUNSA, P.O. Box 215, Pretoria 0204, South Africa; 2 Non-Communicable Diseases Research Unit, South African Medical Research Council (SAMRC), Cape Town 7505, South Africa; 3 Faculty of Health Sciences, University of Limpopo, Polokwane 0700, South Africa

**Keywords:** malnutrition, multilevel factors, school children, mothers, mixed methods, rural context, South Africa

## Abstract

To address childhood malnutrition, the use of multifaceted methodologies, such as mixed methods research, is required to inform effective and contextual interventions. However, this remains limited in studying malnutrition among school children in a South African context, notwithstanding its persistence. We adopted a convergent parallel mixed methods design to best understand the magnitude of malnutrition through multilevel influences in a rural area. A quantitative survey determined the magnitude of malnutrition and associated factors among school children and their mothers (n = 508), parallel to a qualitative study, which explored mothers’ insights into the influences of child growth and nutrition in interviews using seven focus group discussions. Mixed methods integration was achieved through convergence of the quantitative constructs developed from measured variables for malnutrition and related factors with ten emergent qualitative themes using a joint display analysis to compare the findings and generate meta-inferences. Qualitative themes on food unavailability and affordability, poor feeding beliefs and practices, and decision to purchase foods were consistent with the quantified poor socio-demographic status of mothers. Furthermore, the qualitative data explained the high prevalence of undernutrition among children but did not corroborate the high estimated households’ food security in the quantitative survey. The misperceptions of mothers on child growth agreed with limited food knowledge as well as lack of knowledge on child growth gathered during the survey. Moreover, mothers believed that their children were growing well despite the high presence of childhood undernutrition. Mothers further overrated the effectiveness of school feeding programmes in providing healthy food to children as compared to their household food. They reported high incidence of food allergies, diarrhea, and vomiting caused by food consumed at school which resulted in children not eating certain foods. This might have impacted on the nutritional status of children since mothers depended on the school feeding program to provide food for their children. The ambiguity of cultural influences in relation to child growth was evident and substantiated during qualitative interview. Mixed methods integration offered a better understanding of malnutrition from empirical findings on interrelated factors at child, maternal, household, and school levels. This study points to a need for multilevel, informed, and contextual multidimensional interventions to contribute towards addressing childhood malnutrition in South Africa.

## 1. Introduction

The literature documents that the different contexts in which child development takes place range from the primary caregivers, family, and school to the community and the broader context of ethnicity and social values, as indicated by Bronfenbrenner [1]. In addition, UNICEF has described child growth as a complex process characterized by interdependent factors, which involve dietary intake, inadequate health, poverty, household food insecurity, inadequate care and feeding practices, and unhealthy household environment [2]. So, children who come into middle childhood having already accrued significant deficits in growth status experience a slower phase of linear growth during middle childhood [3,4,5,6], resulting from a dynamic relationship of various influences [7]. The health implications of malnutrition pose a serious public health problem, predisposing children to poor mental development, school achievement, and behavioral abnormalities [8,9,10], as well as increased risk of non-communicable diseases (NCDs) later in life [11]. Of note is that the long-lasting effects of undernutrition include an increased susceptibility to central fat accumulation, lower fat oxidation, insulin resistance in adulthood, as well as hypertension and dyslipidaemia [10]. Therefore, considering the multifactorial nature of malnutrition and its consequences [12], investigating childhood malnutrition requires multifaceted methodologies, such as combined quantitative and qualitative approaches, beyond using one method in isolation [13].

Lately, mixed methods research (MMR) has recently been used prominently to investigate complex health-related topics [14], and to analyze the drivers of growth failure [15]. The prominence of mixed-methods lies in its ability to present a framework that draws on the potential strengths of both qualitative and quantitative methods [16], and helps with understanding the complex impacts on interventions in specific contexts [17]. Although MMR provides a better understanding of the research problem than using purely one method alone, the goal is not to replace either quantitative or qualitative research [18]. Moreover, using several data sources to investigate the same phenomenon is advantageous, as in triangulation, which requires the enhancement of the validity of research findings by corroboration, convergence, or correspondence of results from different methods [16,18,19,20]. In addition to triangulation, the purpose of mixing research techniques, methods, and concepts into a single study entails complementarity (i.e., results from one method help to enhance/clarify the results from another method), development (i.e., the results from one method help inform/develop the other methods), initiation (i.e., identifying the contradictions or paradoxes that might reframe the research question), and expansion (i.e., quantitative and qualitative analyses are used to expand the study’s scope and focus) [16].

Several studies in low-and-middle-income countries (LMICs) have used mixed methods to determine nutrition-related issues in populations [13,21,22,23], and few studies in South Africa [24,25,26]. For an example, the use of MMR to investigate malnutrition among children in Madagascar has allowed for consideration of anthropometric data in light of factors, such as family nutrition practices, health behaviors, and household resources, to create a hierarchical framework that provides a better prediction of malnutrition and related health outcomes [21]. In a South African context, a study on the prevention of childhood obesity using MMR reported the usefulness of a combined strategy made up of education, diet, and physical activity, together with the dissemination of food information, which includes addressing the needs of participants, empowering and encouraging decision-making and choice of foods, and changing nutrition attitudes, beliefs and influences based on resources available and within cultural boundaries [24]. Notably, some of the outcomes from mixed analyses on reducing stunting have provided extremely useful insights into the individual, household, community, and macro level drivers of change [27] in several countries, e.g., Bangladesh, Nepal, Senegal, and Zambia [28].

In the context of economic and demographic transition, stunting remains one of the persistent concerns with alarming rates of overweight and obesity in South Africa [29,30,31,32,33,34], giving rise to a double burden of malnutrition [35,36]. This rationalized use of a convergent parallel mixed methods design allows to best understand the magnitude of malnutrition through multilevel influences in a rural area. The significance of this study lies in the fact that the findings might yield comprehensive information necessary to develop multilevel and contextual interventions towards addressing childhood malnutrition in South Africa.

## 2. Methods and Materials

### 2.1. Study Design and Population

This paper summarizes a doctoral thesis (i.e., umbrella study) [37] on growth patterns and societal cultural beliefs and practices in Dikgale, Limpopo Province, a mixed methods study of primary school children and their mothers, approved by the Sefako Makgatho Health Sciences University Research and Ethics Committee, on 4 August 2016, with identification code of SMUREC/H/161/2016: PG. The umbrella study was conceptualized and designed using the Bronfenbrenner model for child development [1] and the UNICEF conceptual framework for malnutrition [2]. The umbrella study consisted of a quantitative component [33,34,38] and a qualitative component [39], of which the results have been published. For this paper, the quantitative and qualitative results were integrated, using a joint display analysis for mixed methods.

The study was conducted in Dikgale Health and Demographic Surveillance System Site (DHDSS), a rural site in Limpopo Province of South Africa, described in detail by Alberts et al. [40]. The site is situated approximately 40 km north-east of Polokwane, the headquarters of Limpopo Province, in South Africa. The total population was approximately 8000 people in 1995 [40], and has been expanded to 15 villages with a population of 36,000 people in 2010. As of 2018, it is called DIMAMO Population Health Research Centre (DIMAMO PHRC) with an approximate population of 100,000 people. DIMAMO PHRC assists the government in planning services, such as health and social, and strengthening the evidence for developing and targeting policies and programmes. The site also assessed demographic dynamics, such as births, deaths, and migrations, as well as important issues and challenges facing the population in areas of NCDs and risk factors, malnutrition and nutrient status, HIV, and lipid profile, including outcome measures for evaluating interventions. Primary schools situated in Dimamo education circuit, mainly from Dikgale area, were used in the study.

### 2.2. Sampling, Data Collection and Analysis

**Figure 1:**
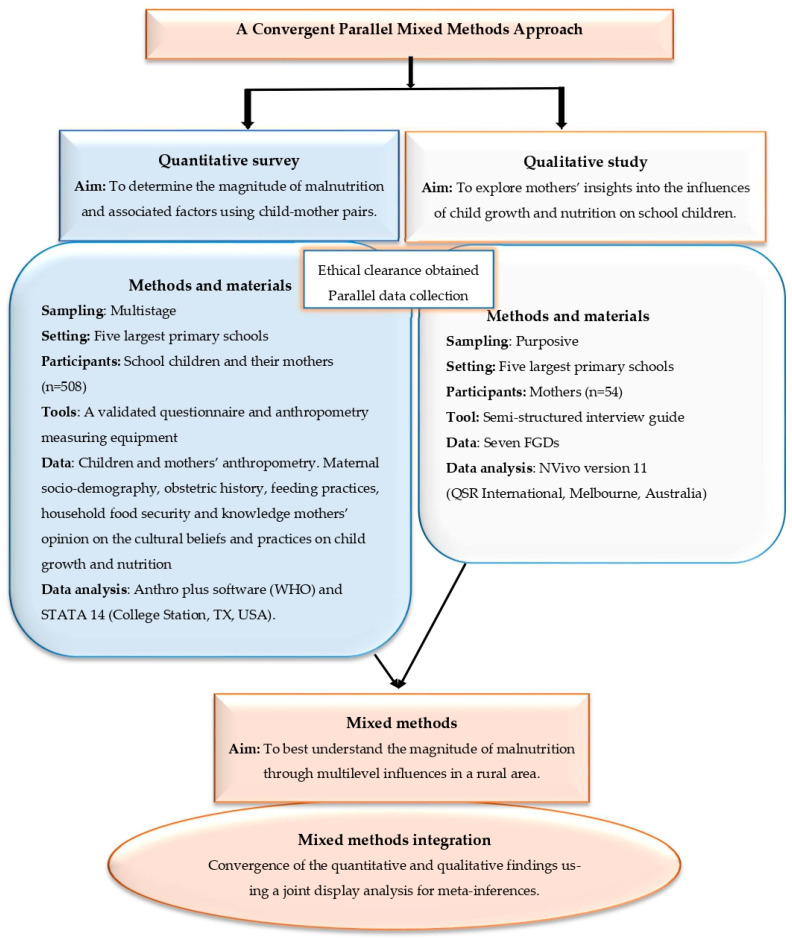
A convergent parallel mixed methods approach to investigate the multidimension of malnutrition among children. Developed by Modjadji and Madiba, 2022.

The methodology used for a quantitative survey and a qualitative study is illustrated in Figure 1, summarizing a convergent parallel mixed methods approach to investigate the multidimension of malnutrition among children.

#### 2.2.1. Quantitative Survey

The quantitative survey determined the magnitude of malnutrition and associated factors among 508 school children paired with their mothers. A multistage sampling technique was used to select children from the five largest primary schools, as per enrolment number and paired with their biological mothers. The study excluded children who were younger than five years, had physical disabilities that compromised their stature, and whose biological mothers were not available to participate. Detailed methodology has been described elsewhere [33,34,38]. In summary, data from the children were collected on age, sex, school grade, and anthropometry (weight and height). A Smart D-quip electronic scale was used to measure weight, while height was measured using a stadiometer, and adhered to the WHO recommendations [41,42]. Anthropometric measurements were converted to height-for-age z scores (HAZ), weight-for-age z scores (WAZ), and body mass index (BMI)-for-age z-scores (BAZ) and compared to reference data for 5–19-year-olds. Children were classified as stunted, underweight, and thin if HAZ, WAZ and BAZ were <−2SD, respectively. Overweight and obesity were categorized as BAZ ≥ +2SD and ≥+3SD, respectively, while z-scores above ≥1SD indicated a possible risk of overweight [42]. A researcher-administered questionnaire was used to collect maternal data on socio-demographic status, obstetric history, household food security, knowledge on child growth and nutrition, and the influences of societal cultural beliefs and practices on child growth. The questionnaire incorporated the determinants of nutritional status and variables mentioned in the BRISK study [2,40,43,44,45], which has been used severally at Dikgale HDSS and yielded reliable results. Content, construct, and face validity were ensured through expertise in the field. Independent translators who speak Sepedi as their mother tongue and are conversant with English did forward and backward translations of the questionnaire. A pilot study was conducted to pre-test the questionnaire and determine its feasibility, including training the research assistants to conduct interviews in Sepedi. Weight (Smart D-quip), height (stadiometer), and waist (WC) and hip (HC) circumferences (non-stretchable tape measure) were measured according to WHO recommendations [41]. Normal weight (BMI: 19.0 to 24 kg/m^2^), underweight (BMI < 18.5 kg/m^2^), overweight (BMI: 25.0 to 29.9 kg/m^2^), obesity (BMI ≥ 30.0 kg/m^2^), and central obesity (WC ≥ 88 cm) and waist-hip ratio (WHR ≥ 0.85) were computed [41]. Descriptive and inferential statistics were performed using STATA version 14 (StataCorp. 2015, Stata Statistical Software: Release 14, College Station, TX, USA).

#### 2.2.2. Qualitative Study

Parallel to the quantitative survey, a qualitative study explored the mothers’ insights into child growth and nutrition in seven FGDs (*n* = 54), consisting of 6–12 members who were selected based on their child being malnourished. Each interview took approximately 60–90 min and was recorded with the consent of the participants. Data collection was informed by data saturation (no new information on the topic of interest could be obtained and when further coding was no longer feasible) [46,47]. A detailed methodology has been described elsewhere [39]. In summary, a semi structured interview guide was consisted of seven open-ended questions covering topics, such as understanding about child growth and nutrition, feeding practices, food security in the households, knowledge on child growth and nutrition, and cultural influences on child growth, which were modified as the data collection proceeded, and follow-up questions and predefined probes were asked in response to the responses given by the participants. Interviews were conducted in Sepedi (a local language), transcribed verbatim from the audio files, and translated into English. Trustworthiness was ensured using several strategies, such as using local language during interviews, a good digital recorder, triangulation, peer debriefing sessions after each FGD, taking field and interview notes, and keeping an audit trail on the process, records, and findings. Transcripts were imported into NVivo QSR version 11 (QSR International, Melbourne, Australia), which was used for data analysis and coding, followed by codebook development through consensual between the researchers on reviewing, refining, and naming of the themes.

## 3. Results

This paper presents the results of the integrated quantitative constructs and qualitative themes in mixed methods design. Combining quantitative and qualitative methods in this study was motivated by the need for triangulation [16,48].

### 3.1. Quantitative Results

The constructs and summary of the main results are given in Table 1. The average age for the children was 10 ± 2 years and for the mothers 37 ± 7 years. Six constructs on malnutrition and related factors were developed, which are (1) anthropometry of children, (2) socio demographic status, (3) obstetric history, (4) household food security, (5) knowledge on nutrition and child growth, and (6) cultural influence. Detailed results have been published [33,34,38]. Undernutrition in children and overweight/obesity in mothers show a household double burden malnutrition amidst poor sociodemographic conditions and obstetric history (constructs 1–3). Factors associated with malnutrition in children and mothers are also indicated in the table. The knowledge of mothers on nutrition and child growth was assessed using questions that required them to provide a “yes”, “no”, or “don’t know” response. Similarly, they were asked several factual questions to assess food security in their household, and answers were “yes”, “no”, and “sometimes”. For these questions, the “yes” results are presented (Table 1).

### 3.2. Qualitative Findings

Ten qualitative themes emerged from the FGDs with the mothers. These were the perceptions on child growth, food unavailability, food affordability, feeding beliefs and practices, decision to purchase foods, perception of mothers about school feeding programme, child food preferences, food knowledge, beliefs and practices during pregnancy, and cultural beliefs and practices on child growth and nutrition. Detailed results are described elsewhere [39]. A summary of the main findings is presented in Table 2.

### 3.3. Mixed Methods Integration

The quantitative results and qualitative findings were analyzed independently, as described above, which is consistent when using a parallel design [49]. Mixed methods integration was achieved through convergence of the six quantitative constructs with the 10 qualitative themes using a joint display analysis to compare the findings and generate meta-inferences.

Table 3 shows the comment column (i.e., meta-inferences), which draws conclusions on the results, indicating whether there is an agreement (congruent) and/or conflict (discordant) between the quantitative constructs and qualitative themes. Results showed that qualitative themes on food unavailability, food affordability, poor feeding beliefs and practices, and decision to purchase foods corresponded with a quantified poor socio-demographic status, and jointly justified a high prevalence of undernutrition among children. The themes further contradicted an overestimated households’ food security in a quantitative survey. The misperceptions of mothers on child growth agreed with limited food knowledge gathered during interviews, and the quantified lack of knowledge on child growth, but contrasted with the presence of childhood undernutrition, since mothers misconceived that their children were growing well. Mothers further misconstrued the benefits of the school feeding programme for the provision of healthy food to children compared to household food, but, on the contrary, cited a habit of food selectivity among children due to experiences of allergies, diarrhea, and vomiting caused by food at school, which may have contributed to poor nutritional status. The ambiguity of quantified cultural influences in relation to child growth was evident and substantiated during qualitative interviews.

## 4. Discussion

This paper aimed to best understand the magnitude of malnutrition through multilevel influences in a rural area using a mixed methods approach. Firstly, the quantitative component of the study showed that childhood undernutrition was prevalent in terms of stunting, underweight, and thinness, and existed with high rates of overweight/obesity among mothers, indicating the presence of a double burden of malnutrition on a household level [33,34]. Most of the factors that were shown to be associated with child undernutrition in the quantitative survey were articulated during the FGDs in the qualitative study.

The context of the study area is rural, and poor socioeconomic status characterized the study population in terms of mothers being single (i.e., never married), with low literacy (i.e., attained primary school education, and did not complete secondary school), unemployed, on minimal household income (i.e., R1000–R5000), and recipients of child support grants. In agreement, the perception of mothers articulated poverty as one of the main drivers of child growth and nutrition in a qualitative strand [39]. Poverty predisposes children to undernutrition through unemployment among caregivers, lack of spousal support, and lack of purchasing power, which ultimately affects food affordability and unavailability, compromising dietary diversity and quality [50,51,52]. The association of childhood undernutrition, particularly stunting, with poor socioeconomic conditions, poor maternal health and nutrition, frequent illness, and/or inappropriate infant and young child feeding and care in early life is documented [53]. Household environment in this study had a poor infrastructure regarding dwelling places and sanitation. Poor living conditions have been reported in the same study area [40,43], and in South Africa at large [54,55].

Mothers perceived socio-economic status and household environment are among other main drivers of child growth and nutrition. Socioeconomic status has the potential to affect nutrition due to the economic barriers that inhibit the ability to buy nutritious food [56]. This was corroborated through qualitative themes on food unavailability and affordability, poor feeding beliefs and practices, and decision to purchase foods consistent with the quantified poor socio-demographic status of mothers. Mothers further narrated the discomfort of food not being enough in their houses and, that low income made it difficult to afford nutritious food and limited their ability to purchase foods. Therefore, they could only afford food, e.g., mealie meal and rice, which affected child feeding. The consumption of energy-dense foods that are not sufficiently nutrient dense to provide the children with adequate nutrition leads to undernutrition, while among mothers these foods promote overweight/obesity [57]. Monotonous diets based mainly on grains originating from energy dense foods lead to both maternal and childhood malnutrition [30,50]. Studies have reported poor feeding practices associated with childhood undernutrition in South Africa, especially with regard to the delayed introduction of solids foods among young mothers [29,58], as well as due to unfavorable sociodemographic status [29,59], which was affirmed in the interviews by mothers in this study. Additionally, poor dietary intake has been reported in the study area [44].

Central to a poverty circumstance established in the current study, the qualitative data explained the high prevalence of undernutrition among children but did not corroborate the high estimated households’ food security in the quantitative survey. Assessing food security entails access to food, food availability, and food utilisation, and in South Africa the consistent emerging trend shows poorer households with women either feeding their children a poor diet or them skipping meals so their children could eat [60]. Regarding food access and availability, poorer households spent less money on food and consumed fewer than eight different food items [60]. Children from households that are food insecure are more likely to have poor growth attainment, recurrent infections, inadequate energy and nutrient intake, compromised learning ability and psychosocial problems [61,62], and all three types of undernutrition (i.e., stunting, underweight, and thinness) are prevalent in this study.

The misperceptions of mothers on child growth agreed with limited food knowledge as well as lack of knowledge on child growth gathered during the survey. Mothers’ nutrition knowledge has been associated with undernutrition in other African countries, e.g., Ghana and Nigeria [63,64]. Mothers’ knowledge of nutrition and health are helpful in safeguarding children from occasions that reduce children HAZ and WHZ scores [64]. However, the most concerning discordance in this study was when mothers believed that their children were growing well despite the high presence of childhood undernutrition. Previous studies have shown that mothers find it difficult to identify the nutritional status of their children appropriately [65,66]. Lack of understanding in which caregivers recognize and respond to children’s linear growth, especially in settings where poor height attainment may not be perceived as abnormal, has been documented [67], as well as in settings with a high prevalence of stunting [68]. Maternal perception influences children’s feeding practices, and affects the quantity and quality of foods offered to children, consequently impacting on their nutritional status [65]. Additionally, maternal poor obstetric history observed in this study is consistent with other studies where mothers with low socioeconomic status are less likely to receive prenatal care [69,70], impacting on child birth growth [71].

The current study also showed poor infrastructure of the schools’ environment in terms of sanitation, documented to be common among schools that are in poorer provinces and in rural areas in South Africa [72,73]. To further describe other main drivers of child growth, mothers further overrated the effectiveness of school feeding programmes in providing healthy food to children as compared to their household food. They reported high incidence of food allergies, diarrhea, and vomiting caused by food consumed at school which resulted in children not eating certain foods. This might have impacted on the nutritional status of children since mothers depended on the school feeding program to provide food for their children. Research has shown that children’s food preferences predict their food consumption patterns [74,75], and, as they grow older, their sphere of environmental influences expands beyond the family to include other environments, e.g., school meal programmes [76]. The school feeding scheme, also known as the National School Nutritional Programme, is the hope of mothers to provide sufficient and quality foods for their children when they are at school. Despite some challenges with NSNP, the program reaches almost all learners, but the school food and nutrition environment are not conducive for promoting healthy eating [77]. Additional reported themes on the mothers’ beliefs and practices during pregnancy, and societal cultural beliefs and practices in the qualitative study [39], minimally showed an intricate relationship between culture and feeding practices. The cultural beliefs and practices in maternal nutrition during the pregnancy or lactating period might continue to influence feeding, including the types of foods children are given because of food taboos, perceptions by mothers, households, and community interactions [78,79,80].

## 5. Strengths and Limitation

The strength of this study lies in the use of mixed methods and integrating the strengths of both the quantitative and qualitative approaches in one study which might have outweighed the weaknesses of both approaches. Although the literature advises caution regarding the validity issue in mixed methods integration, this study has potentially made valid inferences triangulated between quantitative and qualitative methods. We acknowledge that MMR within health care remains an emerging field and its use is subject to much debate. While we used MMR to investigate the multilevel factors influencing malnutrition, we cannot claim to have included all the factors that are related to malnutrition.

## 6. Conclusions

Empirical evidence from this study showed that mixed methods integration offered a better understanding on the magnitude of malnutrition and its multilevel influences. Qualitative themes on food unavailability and affordability, poor feeding beliefs and practices, and decisions to purchase foods were consistent with the quantified poor socio-demographic status of mothers. Qualitative data further explained the high prevalence of undernutrition among children but did not corroborate the high estimated households’ food security in the quantitative survey. The misperceptions of mothers on child growth agreed with limited food knowledge as well as lack of knowledge on child growth gathered during the survey. Moreover, mothers believed that their children were growing well despite the high presence of childhood undernutrition with overrated effectiveness of school feeding programmes in providing healthy food to children as compared to their household food. High incidence of food allergies, diarrhea, and vomiting caused by food consumed at school were reported, which resulted in children not eating certain foods. This might have impacted on the nutritional status of children since mothers depended on the school feeding program to provide food for their children. The ambiguity of cultural influences in relation to child growth was evident and substantiated during qualitative interview. Therefore, MMR integration displayed more congruency between the quantitative constructs and the qualitative themes, except for few discordances.

There is a need for interventions that consider the relevance of the mother’s role and help her recognize the nutritional status of her children appropriately. With limited data of MMR applied in malnutrition studies of children in South Africa, more studies are needed to yield conclusive evidence to continuously improve interventions. This paper provides baseline information for studying the multidimension of malnutrition among children using MMR. Additionally, the comprehensive evidence obtained from this study points to the need for informed and contextual multilevel interventions to contribute towards addressing childhood malnutrition in South Africa.

## Figures and Tables

**Table 1:** Summary of the main quantitative results of children and their mothers with developed six constructs.

Constructs	Main Results
1. Anthropometry	Children: stunting (22%), underweight (24%), thinness (25%); coexistence of stunting and underweight (43%), underweight and thinness (66%). Factors associated with undernutrition: child sex, learning grade, and age, and maternal age, BMI, and short stature, as well as household access to water and using a refrigerator to store food.
	Mothers: mean age (37 ± 7 years), overweight (27%), obesity (42%), combined overweight/obesity (69%). Factors associated with overweight/obesity: age, marital status, living with a spouse as a household head, low household income/month, age of first pregnancy, and more than one pregnancy.
2. Socio demographic status	Mothers: single (63%), unemployment (82%), low literacy (41%), household head (38%). Household: income/month between R1000–R5000 (51%), recipient of child social grant (87%), ≥5 household members (64%), ≥3 children in household (68%), water access (74%), poor sanitation (i.e., using pit toilets) (96%), and using a refrigerator to store food (65%).
3. Obstetric history	Age of first pregnancy (>30 years) (30%), number of pregnancies (2–4) (68%), parity [1 (22%), 2–4 (69%), and 5+ (9%)], and pregnancy complications and outcomes (26%).
4. Household food insecurity	Child eats breakfast every-day (75%), takes lunch box to school every day (48%). Mother worried that child has insufficient food at school (53%) or home (56%), is responsible for purchasing food at home (77%), can buy food for everyday needs (45%), eats less food due to insufficient food (47%), and no food at home sometimes, due to insufficient funds (41%).
5. Knowledge on child growth and nutrition	Mothers believed that: Child is growing according his/her age (82%) Child growth can be affected by number of children a mother has (31%), mother’s health conditions (51%) and sickness (66%), mother’s employment status (44%), number of household members (38%), household income (53%), mother’s feeding knowledge (63%) and practice (93%), feeding them same food (53%), and genetics/heredity (60%).
6. Cultural influence	Societal cultural beliefs and practices influence child growth (37%), mother’s cultural beliefs and practices affect child growth (33%), cultural beliefs influence the way children are fed (24%), there are cultural beliefs that prescribe which food is good for child’s growth (65%), there are cultural belief that prescribe which food is not good for child’s growth (37%), religion influences what to eat and not eat during pregnancy (52%). Most mothers (63%) did not know in which way do cultural beliefs and practices influence child growth.

**Table 2:** Summary of the main findings of the FGDs with mothers with ten emerged themes.

Themes	Main Findings
1. Perception about child growth	Mothers seemed to believe that their children are growing well, but differently. Factors that they considered to affect the growth of their children included socioeconomic status and poverty, genetics, and parents/family physical stature (i.e., heredity from the family), food consumption, maternal feeding practices, and household environment.
2. Food unavailability	Some mothers expressed substantial concerns over food not always being available or enough in the household, with the understanding that food is important for child growth.
3. Food affordability	Mothers narrated in a hypothetical manner that having enough food in their households would entail having the basic foods such as mealie meal flour, sugar, and tea, in addition to fruits and vegetables which they have only when they can afford to it.
4. Feeding beliefs and practices	Mothers indicated the importance of feeding on child growth. Feeding beliefs and practices included early and childhood feeding practices, provision of lunchboxes for school, as well as the types of foods.
5. Decision to purchase food	Most mothers described their buying practices in terms of what informs the food they purchased, in addition to them taking the decision, based on their objective.
6. Perceptions on school feeding programme	Most mothers perceived the food at school to be better than the food at home; they described a variety of foods, such as samp, beans, pap, and milk, that were available at schools compared to their households. Some mothers made suggestions as to how the feeding scheme at schools could be improved to meet the needs of the children.
7. Child food preference	Mothers reported that child eating behaviours such as food preference influence child growth. Mothers described that, children like and dislike certain foods, both at home and at school.
8. Food knowledge	Mothers have limited knowledge on what constitutes healthy food. They were however able to identify nutritious foods that they served their families, such as traditional foods which they believed were good for their children and described healthy foods for child growth.
9. Beliefs and practices during pregnancy	Mothers shared beliefs or practices adopted during pregnancy as expected by their parents or the community; most practices were related to what to eat and not to eat during pregnancy.
10. Societal cultural beliefs and practices	Most mothers did not report any cultural practices that influenced how they raised their children, except for very few ambiguous insinuations.

**Table 3:** Organising the related themes and constructs and creating a joint display analysis.

Qualitative Themes	Quantitative Constructs	Comment (Meta-Inferences)
(1) Perception about child growth	Anthropometry of children. Knowledge on child growth and nutrition.	Discordant—mothers believed that their children were growing well, yet undernutrition was prevalent. Congruent—the perceptions of mothers on child growth were comparable to their lack of knowledge on child growth and nutrition.
(2) Food unavailability	Socio demography. Anthropometry of children. Household food security.	Congruent—food unavailability agreed with the presence of childhood undernutrition and poor socio-demography. Discordant—food unavailability articulated during interviews was not supported by the household food insecurity observed in quantitative analysis.
(3) Food affordability	Anthropometry of children. Socio-demography. Household food security.	Congruent—childhood undernutrition was affirmed by food affordability issues and agrees with poor socio-demography. Discordant—the household food insecurity discussed in the interviews was contrary to quantitative analysis.
(4) Feeding beliefs and practices	Anthropometry of children. Socio-demography. Knowledge on child growth and nutrition. Cultural influence. Household food security.	Congruent—there were agreements on childhood undernutrition with feeding belief and practices, as well as socio demography, knowledge on child growth and nutrition, and to some extent, with cultural influence. Discordant—feeding beliefs and practices reported during interviews were not supported by household food security reported in quantitative analysis.
(5) Decision to purchase food	Anthropometry of children. Socio-demography. Household food security.	Congruent—childhood undernutrition was confirmed by challenges on the decision to purchase foods due to poverty articulated in the interviews and agrees with poor socio-demography. Discordant—decision to purchase foods was not supported by the household food security in the quantitative analysis.
(6) Perceptions on school feeding programme	Anthropometry of children. Knowledge on child growth and nutrition.	Discordant—childhood undernutrition did not agree with mothers’ perceptions on school feeding regarding food to be enough at the schools, and knowledge of mothers on child growth and nutrition.
(7) Child food preference	Anthropometry of children	Congruent—child food preference, mainly at school, where children were selective of foods, they eat due to allergies they experience, suffer from diarrhoea and vomiting, which was confirmed by childhood undernutrition.
(8) Food knowledge	Knowledge on child growth and nutrition. Anthropometry of children	Congruent—food knowledge was confirmed by mothers’ lack of knowledge on child growth and nutrition and agreed with childhood undernutrition.
(9) Beliefs and practices during pregnancy	Obstetric history. Cultural influence	Congruent—beliefs and practices during pregnancy were confirmed by their obstetric complications and, and to some extent, the cultural influence.
(10) Societal cultural beliefs and practices	Cultural influence	Congruent—the ambiguity of the societal cultural beliefs and practices narrated during interviews were substantiated to be unclear in qualitative analysis.

## Data Availability

The dataset and transcripts used and analyzed during the current study are available from the corresponding author on reasonable request.
